# Photophysical Study and Biological Applications of Synthetic Chalcone-Based Fluorescent Dyes

**DOI:** 10.3390/molecules26102979

**Published:** 2021-05-17

**Authors:** Sirilak Wangngae, Kantapat Chansaenpak, Jukkrit Nootem, Utumporn Ngivprom, Sirimongkon Aryamueang, Rung-Yi Lai, Anyanee Kamkaew

**Affiliations:** 1 School of Chemistry, Institute of Science, Suranaree University of Technology, Nakhon Ratchasima 30000, Thailand; swsirilak00@gmail.com (S.W.); utumporn_n@kkumail.com (U.N.); tar1412xa@gmail.com (S.A.); 2 National Nanotechnology Center, National Science and Technology Development Agency, Thailand Science Park, Pathum Thani 12120, Thailand; kantapat.cha@nanotec.or.th (K.C.); jukkrit.noo@ncr.nstda.or.th (J.N.); 3 Center for Biomolecular Structure, Function and Application, Suranaree University of Technology, Nakhon Ratchasima 30000, Thailand

**Keywords:** chalcones, fluorescence imaging, mega-stokes shift dye, bioimaging, antibacterial

## Abstract

A chalcone series (**3a**–**f**) with electron push–pull effect was synthesized via a one-pot Claisen–Schmidt reaction with a simple purification step. The compounds exhibited strong emission, peaking around 512–567 nm with mega-stokes shift (∆λ = 93–139 nm) in polar solvents (DMSO, MeOH, and PBS) and showed good photo-stability. Therefore, **3a**–**f** were applied in cellular imaging. After 3 h of incubation, green fluorescence was clearly brighter in cancer cells (HepG2) compared to normal cells (HEK-293), suggesting preferential accumulation in cancer cells. Moreover, all compounds exhibited higher cytotoxicity within 24 h toward cancer cells (IC_50_ values ranging from 45 to 100 μM) than normal cells (IC_50_ value >100 μM). Furthermore, the antimicrobial properties of chalcones **3a**–**f** were investigated. Interestingly, **3a**–**f** exhibited antibacterial activities against *Escherichia coli* and *Staphylococcus aureus*, with minimum bactericidal concentrations (MBC) of 0.10–0.60 mg/mL (375–1000 µM), suggesting their potential antibacterial activity against both Gram-negative and Gram-positive bacteria. Thus, this series of chalcone-derived fluorescent dyes with facile synthesis shows great potential for the development of antibiotics and cancer cell staining agents.

## 1. Introduction

**Figure 1:**
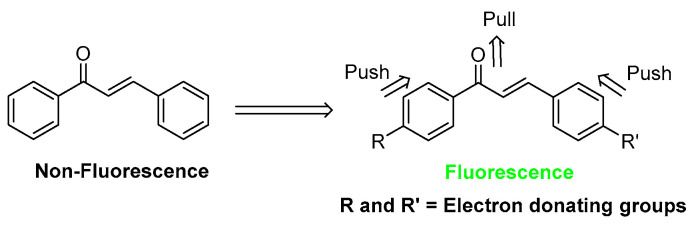
Structural design of chalcone-based fluorescent dyes.

Chalcones, α,β-unsaturated aromatic ketones, constitute the central skeleton of many important biological compounds [1] and are the biosynthetic precursors of flavonoids and isoflavonoids in plants [2,3]. Their diverse structures can cyclize to form various flavonoid compounds with different biological activities [4,5]. In the past decades, many synthetic analogues, such as aza-chalcones and chalcone derivatives containing isoxazole, pyrazole, and indole, [6] were developed and have shown interesting biological properties, such as antioxidant, anticancer, antimicrobial, antiprotozoal, antiulcer, antiviral, antihistaminic, anti-HIV, cytotoxic, and anti-inflammatory activities [7,8,9]. Moreover, chalcones with a suitable electron push–pull arrangement of functional groups were reported to exhibit bright fluorescence [10,11,12,13] (Figure 1), which is applicable for bioimaging. However, their photophysical and photochemical properties have not been systematically investigated for biological applications [14,15].

We are particularly interested in exploring chalcones with fluorescent properties and use them for biological applications, such as cellular imaging and microorganism targeting. To achieve these goals, the development of chalcone-based fluorescent dyes with mega-stokes shifts and high photo-stability is important [16,17]. First, a mega-stokes shift (>100 nm) is an essential factor for bioimaging because it can provide a better signal-to-noise fluorescence [18,19]. Some commonly used fluorescent dyes, such as fluorescein [20], rhodamine [21], cyanine [22], nile red [23], and BODIPY [19,24], exhibit small stokes shifts (∆λ < 70 nm) [20,21], which allow them to re-absorb the emitted photons, leading to undesired background interference [25]. Photo-stability is another important parameter to evaluate for practical bioimaging applications of fluorescent dyes.

Some chalcone-based fluorescent compounds have been evaluated to have good photophysical properties [26,27,28,29], and some showed promising results in biological applications. For example, the diamino-chalcone library was discovered to comprise fluorescent probes for mouse embryonic stem cells, targeting glycoproteins on stem cell surface [13]. Cyclic chalcone analogs of ciproxifan with a tetralone motif were synthesized and evaluated as fluorescent ligands for human histamine H3 receptors (hH3R). The ligand affinities were in the nanomolar concentration range, which opens new possibilities for non-radioactive visualization in pharmacological analysis [30]. In addition, another chalcone-based fluorescent probe (carbazole–chalcone) was synthesized for thiophenol detection. It showed good performance in the analysis of real water samples and living cells, since it showed low cytotoxicity [31].

Previously, the 4-dimethylamino group has been proposed as a good donor group for enhanced fluorescence via push–pull effects in the chalcone structure [32,33,34,35]. However, various functional groups at the other end of the chalcone structure have not yet been systematic studied for their photophysical properties and biological application [35]. The good donor ability of a 4-dialkylamino group on one ring together with suitable electronic properties of the other ring substituents, as well as the planar conformation of the core structure, would be essential for optimal fluorescence.

In this study, a series of chalcone derivatives, **3a**–**f**, was synthesized by one-pot Chaisen–Schmidt reactions using 4-dimethylaminobenzaldehydes and acetophenones with different electron-donating groups at the *para*-position or 2-acetylthiophene. All synthesized chalcone derivatives resulted soluble in phosphate-buffered saline (PBS) containing 3% Tween 80, which is suitable for biological applications. Their fluorescence spectra showed large stokes shifts (∆λ = 93–139 nm), which could be useful for bioimaging. Therefore, the chalcones **3a**–**f** were applied in imaging of normal cells (human embryonic kidney 293, HEK-293) and cancer cells (human hepatocellular carcinoma, HepG2). Both cell lines are commonly used in research because they are easy to handle and suitable for various types of assays [36,37]. Moreover, the antimicrobial properties of **3a**–**f** were also tested against a Gram-negative bacterium (*Escherichia coli*) and a Gram-positive bacterium (*Staphylococcus aureus*), which are common members of the human microbiota but can act as opportunistic pathogens and cause disease.

## 2. Results and Discussion

### 2.1. Synthesis of Chalcones ***3a**–**f***

**Scheme 1:**
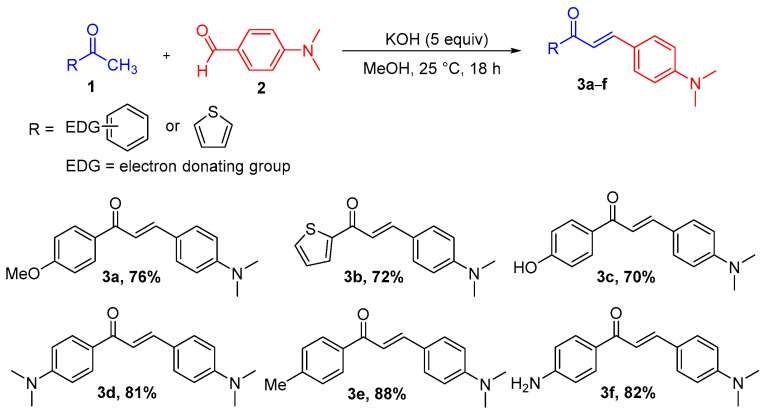
Synthesis of chalcones **3a**–**f** via the Claisen–Schmidt reaction.

The structure of chalcones **3a**–**f** [38,39,40,41] was designed to allow synthesis from various aromatic ketones (**1)** and an aldehyde (**2**), both containing electron-donating groups to push electron towards the carbonyl group of the resulting chalcones. 4-Dimethylaminobenzaldehyde **2** was chosen as the aldehyde, because it was reported to have good donor ability. Furthermore, the substituents of the aromatic ketone **1** varied so to observe their effects on the photophysical properties. The chalcones **3a**–**f** were synthesized via one-pot Claisen–Schmidt reaction (Scheme 1), according to a previously published method [41]. The substrates **1** with various *para*-substituted acetophenone or 2-acetylthiophene moieties were reacted with 4-dimethylaminobenzaldehyde **2** to generate chalcones **3a**–**f** with good yields (70–88%) after filtration, without column purification. The chalcones **3a**–**f** were analyzed by ^1^H-NMR, ^13^C-NMR, and HRMS (in ESI).

### 2.2. Photophysical Properties of Chalcones ***3a**–**f***

**Figure 2:**
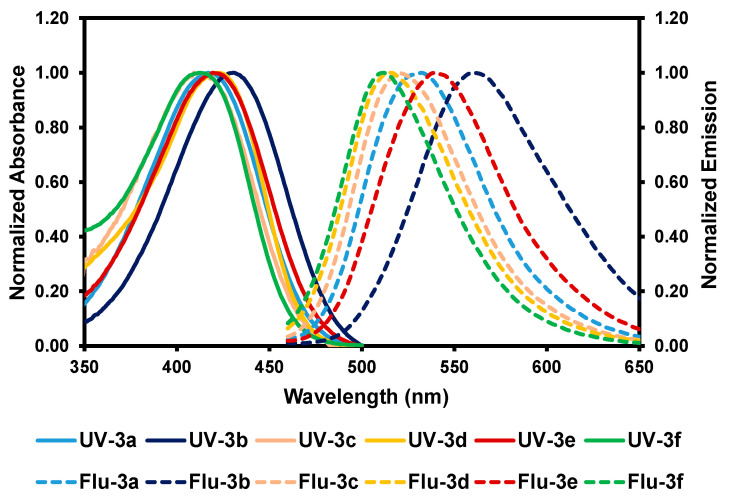
Normalized absorption and fluorescence spectra of chalcones **3a**–**f** in DMSO. Fluorescence spectra with the excitation wavelength at the absorption maximum of each compound.

The absorbance and fluorescence spectra of chalcones **3a**–**f** were measured in DMSO, MeOH, and PBS containing 3% Tween 80. All compounds in DMSO exhibited the maximum absorbance at wavelengths ranging from 412 to 431 nm (Figure 2 and Table 1). Similar absorbance profiles were also observed in the protic solvents MeOH and PBS (Table 1 and Figures S1 and S2 in Supplementary Materials). Notably, all compounds in every solvent showed mega-stokes shifts ranging from 93 to 139 nm, with fluorescent emission wavelengths ranging from 512 to 567 nm. These mega-stokes shifts are beneficial for bioimaging applications because they could reduce self-quenching resulting from molecular self-absorption. Moreover, the fluorescent quantum yields (Φ_f_) of chalcones **3a**–**f** in the polar aprotic solvent (i.e., DMSO) were higher than those in polar protic solvents (i.e., MeOH and PBS).

### 2.3. pH Effects of Chalcones ***3a**–**f*** by Fluorescence Spectroscopic Analysis

**Figure 3:**
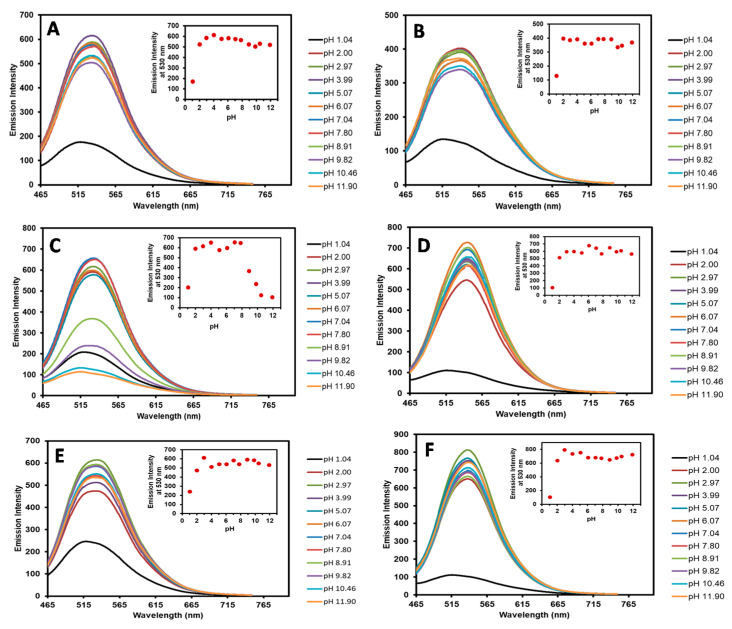
Fluorescence spectra of **3a** (**A**), **3b** (**B**), **3c** (**C**), **3d** (**D**), **3e** (**E**), and **3f** (**F**) in pH 1.04–11.90 buffers.

Since the chalcones **3a**–**f** contain a dimethylamino (-NMe_2_) group that could form different charge states at different pH [42,43], we postulated that this phenomenon could change the photophysical properties of our chalcones. Therefore, to observe changes in the optical property of chalcones **3a**–**f** under different pH, the emission spectra of **3a**–**f** were recorded at various pH values ranging from 1.04 to 11.90 (Figure 3). The spectral analysis indicated that all chalcones (**3a**–**f**) showed decreasing fluorescent signals in highly acidic conditions (pH 1), indicating that the dimethylamino moiety (NMe_2_) was fully protonated. Higher emission peaks were observed over a wide pH range from 2 to 12 for chalcones **3a**, **3b**, **3d**, **3e**, and **3f**, suggesting that the electron push–pull effect from the dimethylamino to the carbonyl group was preserved (Figure 3A,B,D–F). On the other hand, chalcone **3c**, which contains a hydroxyl group (-OH), showed reduced emission signals in basic conditions (pH 9–12) (Figure 3C). This phenomenon could result from the deprotonation of the phenolic proton to generate a phenoxide ion, whose electron can delocalize to the carbonyl group, which would alter the electron flow direction of the compound. Based on these results, all chalcone derivatives sustained their strong fluorescence signals over a wide pH range, which is suitable for cell imaging experiments.

### 2.4. Photostability of Chalcones ***3a**–**f***

**Figure 4:**
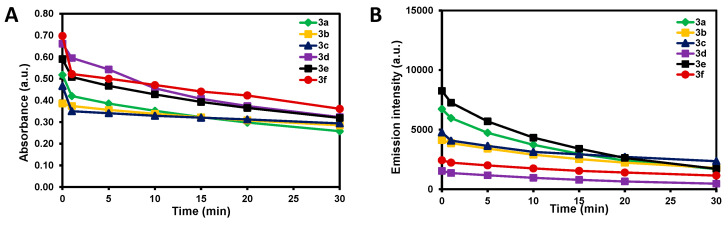
Photobleaching studies at 250 W blue light monitored at the maximum absorbance (**A**) and emission intensity (**B**) of chalcones **3a**–**f** in DMSO.

The photostability of chalcones **3a**–**f** against photobleaching was assessed in DMSO (Figure 4). In this experiment, each chalcone derivative was exposed to irradiation with 250 W blue light (380–500 nm) in an air-saturated condition for 30 min. The photostability was monitored by UV–Vis (Figure 4A) and fluorescence (Figure 4B) spectrophotometry. After 1 min irradiation, the maximum absorbance of all compounds, except for **3b**, dropped dramatically. However, when the exposure time was prolonged from 5 to 30 min, all compounds (**3a**–**f**) showed the minimal loss in the maximum absorbance and emission, suggesting that the compounds are quite stable under blue light irradiation for up to at least 30 min. The decrease in absorbance and fluorescence intensities (% photobleaching) of chalcones **3a**–**f** was also determined based on the difference between the areas under the absorption and emission spectra of chalcones **3a**–**f** before and after photobleaching (Figure S4 in Supplementary Materials). By this method, the calculated percentages of photobleaching of absorbance (after 30 min irradiation, Figure S4A in Supplementary Materials) of **3a**–**f** in DMSO were found to be 50%, 26%, 37%, 51%, 46%, and 48%, respectively. Figure S4B (in Supplementary Materials) shows the calculated % photobleaching of fluorescence (after 30 min irradiation) of **3a**–**f** in DMSO (25%, 43%, 50%, 30%, 21%, and 47%, respectively).

### 2.5. Cell Viability

**Figure 5:**
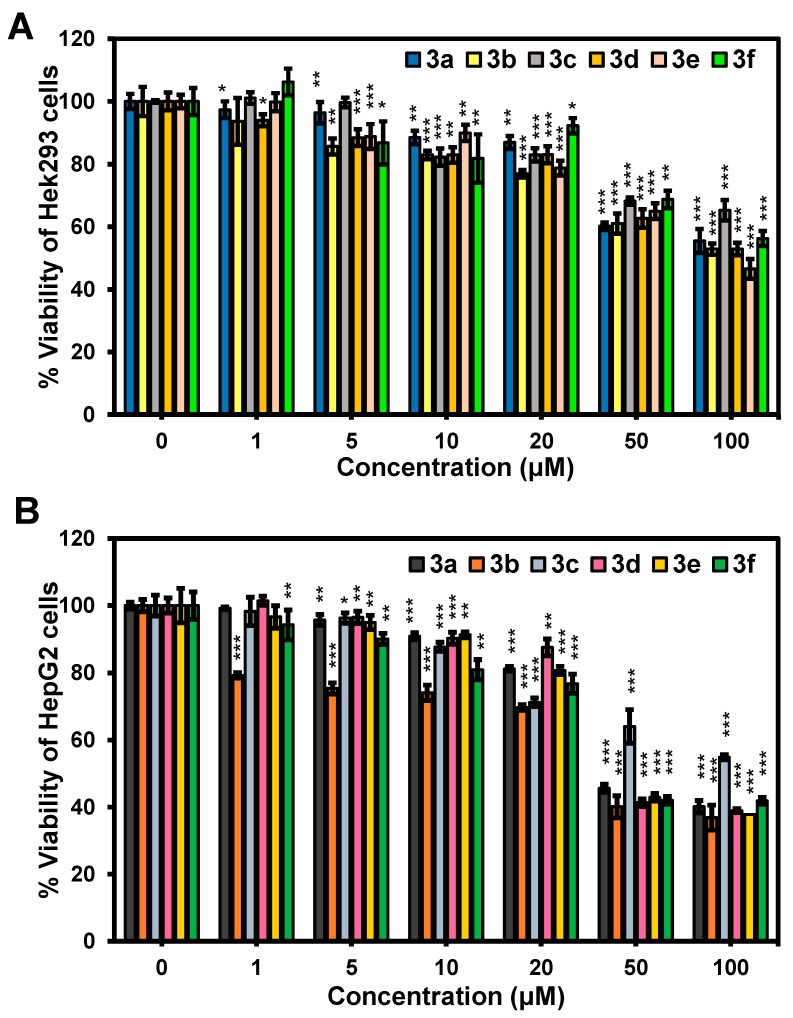
Cytotoxicity effect of chalcones **3a**–**f** on the cellular viability of HEK-293 (**A**) and HepG2 (**B**). All cells were treated with chalcones **3a**–**f** at different concentrations for 24 h (error bar represents standard deviation, *n* = 3). Statistical analysis is based on one-way ANOVA (* *p* < 0.05, ** *p* < 0.01, *** *p* < 0.001).

Next, the cellular toxicity of chalcones **3a**–**f** was tested in two cell lines, i.e., normal (HEK-293) and cancer (HepG2) cells. The cells were treated with various concentrations of **3a**–**f** (0, 1, 5, 10, 20, 50, and 100 μM) for 24 h. The comparative cell viability was determined by MTT assays. As shown in Figure 5A, the normal cells retained more than 80% viability at concentrations up to 20 μM, whereas at the high concentration (50 μM), the viability of the cells dropped to about 60%. For the cancer cells, similar cytotoxicity profiles of **3a**–**f** were observed. However, the cell viability was dramatically reduced to about 40% at the concentration of 50 μM (Figure 5B). In addition, the chalcones (**3a**–**f**) were determined to have IC_50_ values of 45–100 µM for HepG2 and >100 µM for HEK-293 cells (Table 2 and Figure S6 in Supplementary Materials). Therefore, the examined cancer cells seem to be more sensitive to our tested compounds than the normal cells, as the IC_50_ values were about 2–3 times lower for each compound. These results are comparable with those of previous anti-cancer activity studies of chalcone series, some of which showed good activity towards cancer cells [38,44,45].

### 2.6. Confocal Imaging

**Figure 6:**
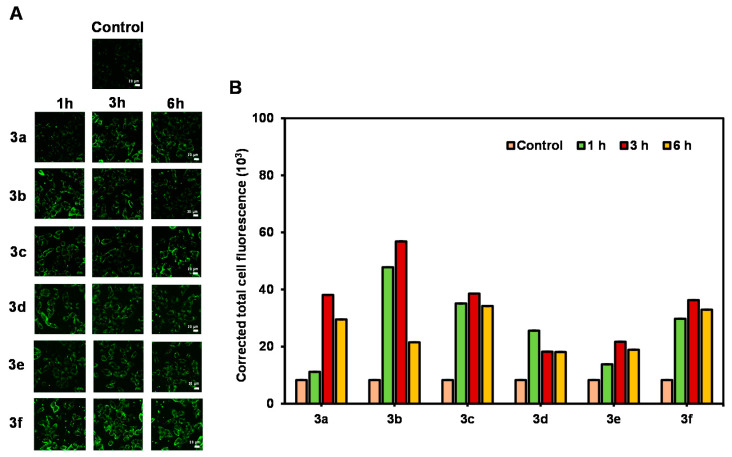
(**A**) CLSM images of HepG2 cells incubated with 5 μM of chalcones **3a**–**f** for 1–6 h. Scale bar = 20 µm. (**B**) Corrected total cell fluorescence data quantified using ImageJ and represent the mean ± SD (*n* = 30).

As **3a**–**f** exhibited bright fluorescence with mega-strokes shifts, their application in live cell imaging was explored. The cancer cells HepG2 were incubated with **3a**–**f** (5 μM) for 1–3 h and then washed thoroughly with PBS before being imaged by a confocal laser scanning microscope (CLSM). The cells incubated with chalcones **3a**–**f** exhibited bright green fluorescence at every time point compared with the control cells (no compound added, Figure 6). The imaging was also performed with the normal cells (HEK-293) using the same treatment. However, the quantitative data did not show significant difference between the green emission from the compounds and that from cell autofluorescence (Figure S5 in Supplementary Materials). In summary, these results suggest that our chalcone derivatives could be used as cancer cell staining probes.

### 2.7. Antibacterial Activity of the Synthesized Chalcones (***3a**–**f***)

**Figure 7:**
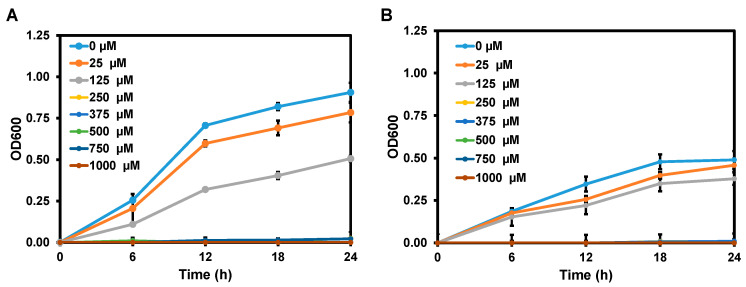
Growth curves of *E. coli* 780 (**A**) and *S. aureus* 1466 (**B**) in response to **3d** in a time course of 24 h.

Lastly, the antibacterial activities of chalcones **3a**–**f** were evaluated in Gram negative bacteria (*E. coli* 780) and Gram-positive bacteria (*S. aureus* 1466). The MIC and MBC values of chalcone **3a**–**f** for *E. coli* 780 ranged from 250 to 375 μM and from 375 to 1000 μM, respectively (Table 3). For *S. aureus* 1466, the MIC values of **3a**–**f** ranged from 250 to 375 μM, which is similar to that for *E. coli* 780 (Table 3), whereas the MBC values were much higher (1000 μM). The greater antibacterial activities of chalcones against *E. coli* 780 than *S. aureus* 1466 might be due to the thinness of the peptidoglycan layer in Gram-negative bacteria [28,46,47,48]. According to the MIC and MBC values, **3d** showed the best antimicrobial activities against *E. coli* 780 and *S. aureus* 1466 compared to the other compounds among the series. Therefore, the growth curves of *E. coli* 780 and *S. aureus* 1466 in the presence of various concentrations of **3d** were obtained by optical density at 600 nm (OD600) for 24 h (Figure 7). As the concentration of **3d** increased, the growth of both bacteria strains was significantly inhibited. The data of all antibacterial experiments are presented in Figures S7 and S8 in Supplementary Materials. The inhibitory results are not as good as those previously reported for chalcones with extra modifications [46]. 

The recent findings on structure–activity relationships (SAR) in medicinal chemistry showed that halogenated chalcones (i.e., fluorine or chlorine-substituted chalcones) have better antibacterial activity. Moreover, some hybrid structures, such as ferrocene–chalcone hybrids or steroidal–chalcone hybrids also exhibit potent antibacterial activities. Alternatively, replacing a 4-dimethylamino group with other electron-donating groups, such as -OH or -OMe, or with electron-withdrawing groups (-Cl, -Br, -I, or -NO_2_), as well as other heterocyclic substituents, would alter the antibacterial activity [46]. These findings suggest that additional structural optimization of chalcone derivatives is necessary for the development of potential antimicrobial drugs.

## 3. Materials and Methods

### 3.1. Material and Instrumentation

The ^1^H- and ^13^C-NMR data were collected on a Bruker 500 MHz NMR (Bruker Ltd., Rheinstetten, Germany) in CDCl_3_, DMSO-*d*_6_, and acetone-*d*_6_ as solvents at room temperature, with tetramethylsilane (TMS) as an internal reference. High-resolution mass spectra (HRMS) were recorded by an electrospray ionization mass (ESI-MS) spectrometer (MicrOTOF, Bruker, Rheinstetten, Germany). The absorbance and fluorescence spectra were measured on a UV–Vis spectrophotometer (Agilent Technologies Cary 300, CA, USA) and a fluorescence spectrophotometer (PerkinElmer LS55, MA, USA), respectively. All glassware was oven-dried prior to use. All reagents and solvents were purchased from the companies Sigma Aldrich and Merck (MO, USA), TCI (Tokyo, Japan), or Carlo Erba (Barcelona, Spain)and used without further purification. Analytical thin-layer chromatography (TLC) was performed on TLC Silica gel 60 F254 (Merck, MO, USA) and visualized under a UV cabinet lamp.

### 3.2. Synthesis

The general synthetic procedure for chalcones **3a**–**f** is shown in Scheme 1. A substituted aromatic/heteroaromatic acetophenone (**1**) (0.67 mmol) was mixed with 4-dimethylaminobenzaldehydes (**2**) (100 mg, 0.67 mmol) in MeOH (2 mL) containing KOH (188 mg, 3.35 mmol). The mixture was stirred for 18 h at 25 °C to yield a precipitate as a product. The solid was filtered out, washed with cold MeOH (20 mL), and finally dried under vacuum. The pure products were obtained as the corresponding chalcones (**3a**–**f**) in 70–88% yield.

### 3.3. Photophysical Properties

All UV–Vis absorption and fluorescence spectra were recorded on a UV–Vis Spectrophotometer (Agilent Technologies Cary 300, Santa Clara, CA, USA) and a Spectrofluorometer (PerkinElmer LS55, Waltham, MA, USA), respectively. In both experiments, the stock solutions of chalcones **3a**–**f** were prepared as 0.5 mM in DMSO. The appropriate amount of the stock solution was added to DMSO, MeOH, and 0.01 M PBS buffer (pH 7.4) with 3% Tween 80 (3 mL) to obtain the final concentration of 2 µM in the testing solutions. For fluorescence experiments, the emission spectra were recorded at the maximum absorbance wavelength of each compound. The fluorescent quantum yields (Φ_f_) were calculated relative to fluorescein in 0.1 M NaOH as a standard (Φ_f_ = 0.95).

### 3.4. Study of the Effect of pH 

Chalcones **3a**–**f** (2 µM) were dissolved in commercial pH 1–12 buffers purchased from Merck (St. Louis, MO, USA) (glycine/NaCl/HCl for pH 1, citric acid/NaOH/HCl for pH 2–6, Na_2_HPO_4_/KH_2_PO_4_ for pH 7, boric acid/ KCl/NaOH for pH 8–11, Na_3_PO_4_/NaOH for pH 12) in the presence of Tween 80 (3% *w*/*w*). The actual pH values of the final buffer solutions were measured by a pH meter. The resulting solutions were examined by a Spectrofluorometer (PerkinElmer LS55) at the maximum absorbance wavelength of each compound.

### 3.5. Photo-Stability Test

Chalcones **3a**–**f** were dissolved in DMSO at the final concentration of 1 × 10^−6^ M for measuring absorption and emission intensity. Then, **3a**–**f** were irradiated at 250 W (blue-lamp) at a distance of 75 cm. The UV–Visible absorption and fluorescence spectra of the solutions were measured at different time points including 0, 1, 5, 10, 15, 20, and 30 min. Photo-stability was reported in terms of photobleaching absorption and emission (%) calculated from the change of absorption and emission intensity at the absorption and emission maxima before and after irradiation.

### 3.6. Confocal Imaging

For fluorescence imaging of living cells, human embryonic kidney 293 (HEK-293, ATCC) and human hepatoma cancer cell (HepG2, ATCC) were cultured in Dulbecco′s Modified Eagle′s Medium (DMEM, HyClone) supplemented with 10% Fetal Bovine Serum (FBS, Gibco, Carlsbad, CA, USA) and 1% Penicillin/Streptomycin Solution 100X (Corning, Corning, NY, USA). All cells were cultured in a humidified incubator at 37 °C with 5% CO_2_. HEK-293 and HepG2 cells, approximately 1 × 10^4^ cells, were seeded on 8-well chambered cover glasses (LabTek, Nunc, Carlsbad, CA, USA) and incubated in complete medium for 24 h. After that, the cells were treated with 5 μM stock solutions of **3a**–**f** in DMSO/cell culture medium containing 10% FBS (0.25% DMSO), for 0, 1, 3, and 6 h. Then, the cells were washed twice with 0.01 M PBS before being visualized under a Laser-Scanning Confocal Microscope (Nikon A1Rsi) with a 60× oil immersion objective lens and living cell workstation with the excitation channel at 488 nm.

### 3.7. Cell Viability Assay

The cells were seeded on a 96-well plate, approximately 7 × 10^3^ cells per well, and incubated in complete medium for 24 h. After that, the cells were treated with 0, 1, 5, 10, 20, and 50 μM of chalcones **3a**–**f**, and culturing continued for 24 h. After incubation, the cells were washed three times with 10 mM PBS and treated with 20 mL of methylthiazolyldiphenyl-tetrazolium bromide (MTT reagent, 0.5 mg mL^−1^, Sigma-Aldrich, St. Louis, MO, USA) for 2–3 h. After medium removal, DMSO was added to dissolve the formazan product, and cell viability was determined by UV–Vis absorption of the resulting formazan at a wavelength of 560 nm in a microplate reader (BMG Labtech/SPECTROstar Nano, Ortenberg, Germany).

### 3.8. Antibacterial Activity Test

All bacteria strains were obtained from the Thailand Institute of Scientific and Technological Research (TISTR). The antibacterial activity of chalcones **3a**–**f** was determined by the minimal inhibitory concentration (MIC) and the minimal bactericidal concentration (MBC) against the Gram-positive (*S. aureus* 1466, TISTR 1466) and Gram-negative (*E.coli* 780, TISTR 780) bacteria [23]. The stocks of chalcones **3a**–**f** (0.60 mg/mL) were serially diluted in Muller–Hinton (MH) broth to obtain concentrations in the range of 250–1000 µM. The solutions of chalcones **3a**–**f** (100 µL) at different concentrations were added in the culture of the tested bacteria (100 µL) at a concentration of 1 × 10^6^ colony-forming units/mL (CFU/mL) in MH broth at 37 °C with shaking at 80 rpm. The bacterial numbers were determined by measuring the optical density at 600 nm by a UV–Vis spectrometer. The MIC value was determined at the minimal concentration of the compounds that completely inhibited bacterial growth. To determine the MBC values, the bacterial cultures (10 µL) containing the solutions of chalcones **3a**–**f** at MIC and three higher concentrations were dropped on MH agar plates. After incubating at 37 °C for 24 h, the MBC value was determined at the minimal concentration of these compounds that killed 100% of the bacterial populations.

## 4. Conclusions

In summary, we found that chalcones containing a 4-dimethylamino group as a strong electron donor moiety combined with the effect of an electron donor from another aromatic end showed enhanced fluorescent emission via push–pull effects. Their photophysical properties were systematically investigated, revealing that the electron push–pull arrangement in the structures caused UV-Vis absorbance at 350–500 nm and fluorescent emission in the visible region (470–700 nm) with mega-stokes shifts (>100 nm). In addition, the chalcone series exhibited high photo-stability and stability in a wide range of pH, which makes them suitable for biological applications. Therefore, their application in bioimaging and in the analysis of anticancer and antibacterial activities was tested. Chalcones **3a**–**f** showed significantly higher cytotoxicity against cancer cells (HepG2) than normal cells (HEK-293), with IC_50_ ranging from 45 to 100 µM and >100 µM, respectively. Moreover, **3a**–**f** could be used as cancer cell-staining probes that emit green light within a few hours of incubation. Furthermore, the synthesized chalcones exhibited antibacterial activity against both *E. coli* and *S. aureus*, suggesting their potential antimicrobial applications after additional chemical modifications. Finally, this study provides a good example of how to synthesize skeleton analogues of natural products through easy steps to generate compounds that possess fluorescence properties for biological applications.

## Figures and Tables

**Table 1:** Photophysical properties of chalcone **3a**–**f** (2 µM) in different solvents.

Solvents	Dye	Photophysical Properties				
		λ_abs_ ^a^ (nm)	λ_em_ ^b^ (nm)	∆λ ^c^ (nm)	Φ_f_ ^d^ (*n* = 3)	ε ^e^ (M^−1^ cm^−1^)
DMSO	**3a**	417	532	115	0.69 ± 0.04	4.6 × 10^4^
	**3b**	431	560	129	0.34 ± 0.04	3.1 × 10^4^
	**3c**	413	520	107	0.61 ± 0.04	1.0 × 10^4^
	**3d**	422	516	94	0.10 ± 0.02	4.6 × 10^4^
	**3e**	420	539	119	0.50 ± 0.02	3.2 × 10^4^
	**3f**	412	512	100	0.21 ± 0.03	3.5 × 10^4^
MeOH	**3a**	417	549	132	0.013 ± 0.006	3.0 × 10^4^
	**3b**	428	567	139	0.012 ± 0.007	2.9 × 10^4^
	**3c**	414	546	132	0.017 ± 0.011	1.0 × 10^4^
	**3d**	423	555	132	0.024 ± 0.015	3.7 × 10^4^
	**3e**	417	547	130	0.012 ± 0.007	3.2 × 10^4^
	**3f**	414	549	135	0.027 ± 0.011	3.1 × 10^4^
PBS (3%*w*/*w* tween 80)	3a	416	529	113	0.15 ± 0.02	3.3 × 10^4^
	3b	427	520	93	0.11 ± 0.02	3.7 × 10^4^
	3c	411	526	115	0.11 ± 0.02	1.3 × 10^4^
	3d	422	542	120	0.17 ± 0.03	4.4 × 10^4^
	3e	420	521	101	0.18 ± 0.03	2.8 × 10^4^
	3f	412	524	112	0.18 ± 0.02	3.4 × 10^4^

^a^ λ_abs_ = absorption maximum wavelength, ^b^ λ_em_ = emission maximum wavelength (excited at the maximum absorption wavelength), ^c^ ∆λ = stokes shifts (λ_em_ − λ_abs_), ^d^ Φ_f_ = fluorescence quantum yields calculated by using fluorescein in 0.1 M NaOH as a standard (Φ_f_ = 0.95). ^e^ ε = molar absorptivity.

**Table 2:** IC_50_ of the chalcones (**3a**–**f**).

Cell Lines	IC_50_ (μM)					
	3a	3b	3c	3d	3e	3f
**HEK-293**	>100	>100	>100	>100	96	>100
**HepG2**	56	45	100	56	54	54

**Table 3:** Antibacterial activities of the compounds **3a**–**f**.

Compounds	*E. coli* 780		*S. aureus* 1466	
	MIC (μM)	MBC (μM)	MIC (μM)	MBC (μM)
**3a**	375 ± 0.01	750 ± 0.02	375 ± 0.03	1000 ± 0.09
**3b**	250 ± 0.02	750 ± 0.02	375 ± 0.01	1000 ± 0.01
**3c**	250 ± 0.01	750 ± 0.01	375 ± 0.01	1000 ± 0.01
**3d**	250 ± 0.01	375 ± 0.01	250 ± 0.01	1000 ± 0.01
**3e**	375 ± 0.01	1000 ± 0.02	375 ± 0.03	1000 ± 0.02
**3f**	375 ± 0.01	750 ± 0.01	375 ± 0.01	1000 ± 0.04

MIC and MBC values are indicated as the mean ± SD (standard deviation) of three independent experiments.

## Data Availability

Data are contained within the article or Supplementary Materials.

## Supplementary Materials

The followings are available online. All the synthetic procedures for compounds **3a**–**f**, Figure S1: Absorption and fluorescence spectra of **3a**–**f** in PBS and MeOH, Figure S2: The UV–Vis absorption and fluorescence spectra of **3b**, **3d**, **3e**, and **3f** at different pH (3, 5, 7, 10, and 12), Figure S3; Absorption and fluorescence spectra of fluorescein in 0.1 M NaOH, Figure S4: Calculated % photobleaching of chalcones **3a**–**f** in DMSO based on absorbance and fluorescence changes, Figure S5: CLSM images of HEK-293 cells incubated with 5 μM of chalcone **3a**–**f** for 1–3 h, Figure S6; Plots of concentrations of **3a**–**f** vs. percent viability of HEK-293 (**A**) and HepG2 (**B**) cells, Figure S7: Growth curves of *E. coli* 780 in response to **3a**–**f** in a time course of 24 h, Figure S8: Growth curves of *S. aureus* 1466 in response to **3a**–**f** in a time course of 24 h. References [38,39,40,41,49] are cited in the Supplementary Materials.

## Sample Availability

Samples of the compounds **3a**–**f** are available from the authors.
