# Supplementary material for: Photophysical Study and Biological Applications of Synthetic Chalcone-Based Fluorescent Dyes
Source: Molecules. 2021 May 17;26(10):2979. doi: 10.3390/molecules26102979 (PMC8156934; doi:10.3390/molecules26102979)
Supplement: Supplementary file 1 [file molecules-26-02979-s001.zip › molecules-1213941-supplementary.pdf]

Supporting Information

Photophysical Study and Biological Applications of Synthetic Chalcone-based Fluorescent Dyes

Sirilak Wangngae[1], Kantapat Chansaenpak[2], Jukkrit Nootem[2], Utumporn Ngivprom [1,3], Sirimongkol Aryamuang [1], Rung-Yi Lai [1, 3,*] and Anyanee Kamkaew [1,*]

[1]School of Chemistry, Institute of Science, Suranaree University of Technology, Nakhon Ratchasima, Thailand 30000.

[2]National Nanotechnology Center, National Science and Technology Development Agency, Thailand Science Park, Pathum Thani, Thailand 12120.

[3]Center for Biomolecular Structure, Function and Application, Suranaree University of Technology, Nakhon Ratchasima, Thailand 30000.

[*]Correspondence: rylai@sut.ac.th and anyanee@sut.ac.th

Table of contents

Experimental Section……………………………………………………………...2

General procedure for the preparation of chalcones (3a-f) …………………………….2

^{1}H NMR, ^{13}C NMR, and MS results of compound 3a-f……………………………........2

Spectrum NMR of chalcones (3a-f) ………………………….. 4

Absorption and fluorescence spectroscopic analyses……………………………………10

Photostability of chalcones 3a-f…………………………………………………………...13

Confocal imaging of chalcones (3a-f) in HEK-293………………………………………14

IC_{50} plots of chalcones (3a-f) for HEK-293 and HepG2 ……………………………… 14

Antibacterial activity of the synthesized chalcones (3a-f)……………………………….15

Experimental Section

Under otherwise noted, materials were obtained from commercial suppliers and used without further purification. Thin layer chromatography (TLC) was performed using silica gel 60 F254 and visualized under UV light. Column chromatography was performed with silica gel (mesh 300-400). ^{1}H NMR and ^{13}C NMR spectra were recorded on a Bruker Avance 500 MHz spectrometer in $CDCl_3$, DMSO-d_6 and acetone-d_6 with Me_4Si as an internal standard. Data were reported as follows: chemical shift in ppm (δ), multiplicity (s = singlet, d = doublet, t = triplet, q = quartet, br = broad and m = multiplet), coupling constant in Herts (Hz) and integration and only major peaks are reported in cm^{-1}. HRMS and mass data were recorded by ESI on a TOF mass spectrometer.

General procedure for the preparation of chalcones (3a-f)

To a mixture of acetophenone **1** (0.67 mmol) and 4-dimethylaminobeazaldehyde **2** (0.67 mmol) in MeOH (2 mL) was added KOH (3.35 mmol). The resulting mixture was stirred for 18 h at room temperature. After that, the yellow precipitate was filtered off, washed with ice cold MeOH (20 mL), and dried under vacuum. The crude product was obtained as the corresponding chalcones (**3a-f**).

1. ^{1}H NMR, ^{13}C NMR, and MS results of compound 3a-f

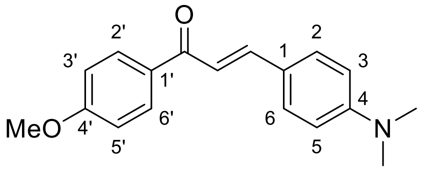

(*E*)-3-(4-(dimethylamino)phenyl)-1-(4-methoxyphenyl)prop-2-en-1-one[1]

141.27 mg, 75% yield; ^{1}H NMR (500 MHz, $CDCl_3$) δ 8.00 (d, J = 9.0 Hz, 2H, C-2' and 6'-H), 7.76 (d, J = 15.5 Hz, 1H, Ar-CH=), 7.50 (d, J = 9.0 Hz, 2H, C-2 and 6-H), 7.32 (d, J = 15.5 Hz, 1H, CO-CH=), 6.93 (d, J = 9.0 Hz, 2H, C-3' and 5'-H), 6.64 (d, J = 9.0 Hz, 2H, C-3 and 5-H), 3.83 (s, 3H, -OCH_3), 2.98 (s, 6H, -$(CH_3)_2$), ^{13}C NMR (125 MHz, $CDCl_3$) δ 188.9, 163.0, 151.9, 144.9, 131.9, 130.6, 130.3, 122.9, 116.7, 113.7, 111.9, 55.5, 40.1, HRMS (ESI) calcd for $C_{18}H_{19}N_1Na_1O_2$ $[M+Na]^+$ 304.1308, found 304.1316.

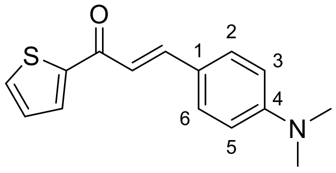

(*E*)-3-(4-(dimethylamino)phenyl)-1-(thiophen-2-yl)prop-2-en-1-one[2]

124.02 mg, 72% yield; ^{1}H NMR (500 MHz, $CDCl_3$) δ 7.76 (d, J = 15.5 Hz, 1H, Ar-CH=), 7.74 (d, J = 3.0 Hz, 1H), 7.53 (d, J = 5.0 Hz, 1H), 7.46 (d, J = 9.0 Hz, 2H, C-2 and 6-H), 7.15 (d, J = 15.5 Hz, 1H, CO-CH=), 7.07 (t, J = 4.5 Hz, 1H), 6.61 (d, J = 9.0 Hz, 2H, C-3 and 5-H), 2.95 (s, 6H, -$(CH_3)_2$), ^{13}C NMR (125 MHz, $CDCl_3$) δ 182.2, 152.1, 146.4, 145.0, 132.9, 130.9, 130.5, 128.1, 122.5, 116.3, 111.9, 40.2, HRMS (ESI) calcd for $C_{15}H_{15}N_1Na_1O_1S_1$ $[M+Na]^+$ 280.0767, found 280.0762.

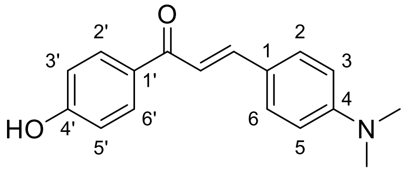

(*E*)-3-(4-(dimethylamino)phenyl)-1-(4-hydroxyphenyl)prop-2-en-1-one[3]

125.28 mg, 70% yield; ^{1}H NMR (500 MHz, Acetone-d_6) δ 9.09 (s, 1H, -OH), 8.03 (d, J = 9.0 Hz, 2H, C-2' and 6'-H), 7.69 (d, J = 15.5 Hz, 1H, Ar-CH=), 7.63 (d, J = 9.0 Hz, 2H, C-2 and 6-H), 7.57 (d, J = 15.5 Hz, 1H, CO-CH=), 6.94 (d, J = 9.0 Hz, 2H, C-3' and 5'-H), 6.76 (d, J = 9.0 Hz, 2H, C-3 and 5-H), 3.03 (s, 6H, -$(CH_3)_2$), ^{13}C NMR (125 MHz, Acetone-d_6) δ 187.0, 161.4, 152.1, 143.9, 131.0, 130.6, 130.2, 122.9, 116.4, 115.1, 111.9, 39.3, HRMS (ESI) calcd for $C_{17}H_{17}N_1Na_1O_1$ $[M+Na]^+$ 290.1151, found 290.1153.

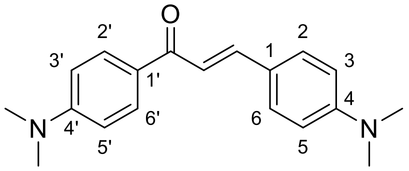

(*E*)-1,3-bis(4-(dimethylamino)phenyl)prop-2-en-1-one[4]

159.65 mg, 81% yield; ^{1}H NMR (500 MHz, $CDCl_3$) δ 8.00 (d, J = 9.0 Hz, 2H, C-2' and 6'-H), 7.77 (d, J = 15.5 Hz, 1H, Ar-CH=), 7.55 (d, J = 9.0 Hz, 2H, C-2 and 6-H), 7.41 (d, J = 15.5 Hz, 1H, CO-CH=), 6.70 (d, J = 9.0 Hz, 4H, C-3', 5'-H and C-3, 5-H), 3.07 (s, 6H, -$(CH_3)_2$), 3.03 (s, 6H, -$(CH_3)_2$), ^{13}C NMR (125 MHz, $CDCl_3$) δ 188.1, 153.1, 151.6, 143.4, 130.6, 130.0, 126.7, 123.4, 117.2, 112.0, 110.8, 40.1, HRMS (ESI) calcd for $C_{19}H_{23}N_2O_1$ $[M+H]^+$ 295.1805, found 295.1804.

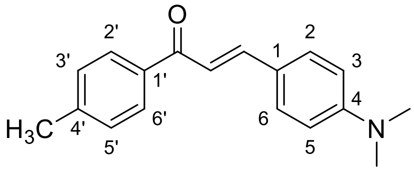

(*E*)-3-(4-(dimethylamino)phenyl)-1-(p-tolyl)prop-2-en-1-one[1]

156.33 mg, 88% yield; ^{1}H NMR (500 MHz, $CDCl_3$) δ 8.03 (d, J = 9.0 Hz, 2H, C-2' and 6'-H), 7.89 (d, J = 15.5 Hz, 1H, Ar-CH=), 7.64 (d, J = 9.0 Hz, 2H, C-2 and 6-H), 7.44 (d, J = 15.5 Hz, 1H, CO-CH=), 7.38 (d, J = 9.0 Hz, 2H, C-3' and 5'-H), 6.78 (d, J = 9.0 Hz, 2H, C-3 and 5-H), 3.12 (s, 6H, -$(CH_3)_2$), 2.52 (s, 3H, -CH_3), ^{13}C NMR (125 MHz, $CDCl_3$) δ 190.2, 152.0, 145.4, 142.9, 136.5, 130.4, 129.2, 128.5, 122.8, 116.9, 111.9, 40.2, 21.7, HRMS (ESI) calcd for $C_{18}H_{20}N_1O_1$ $[M+H]^+$ 266.1539, found 266.1599.

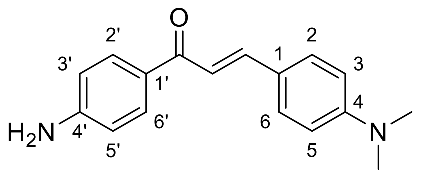

(*E*)-1-(4-aminophenyl)-3-(4-(dimethylamino)phenyl)prop-2-en-1-one[5]

146.22 mg, 82% yield; ^{1}H NMR (500 MHz, DMSO-d_6) δ 7.96 (d, J = 8.5 Hz, 2H, C-2' and 6'-H), 7.72 (d, J = 8.5 Hz, 2H, C-2 and 6-H), 7.66 (d, J = 15.5 Hz, 1H, Ar-CH=), 7.62 (d, J = 15.5 Hz, 1H, CO-CH=), 6.80 (d, J = 8.5 Hz, 2H, C-3' and 5'-H), 6.68 (d, J = 8.5 Hz, 2H, C-3 and 5-H), 6.11 (s, 2H, -NH_2), 3.06 (s, 6H, -$(CH_3)_2$), ^{13}C NMR (125 MHz, DMSO-d_6) δ 186.3, 153.8, 152.0, 142.9, 131.2 130.6, 126.4, 123.0, 117.1, 113.2, 112.3, 40.2, HRMS (ESI) calcd for $C_{17}H_{19}N_2O_1$ [M+H]$^+$ 267.1492, found 267.1499.

2. Spectrum NMR of chalcones (3a-f)

^{1}H NMR of compound **3a**

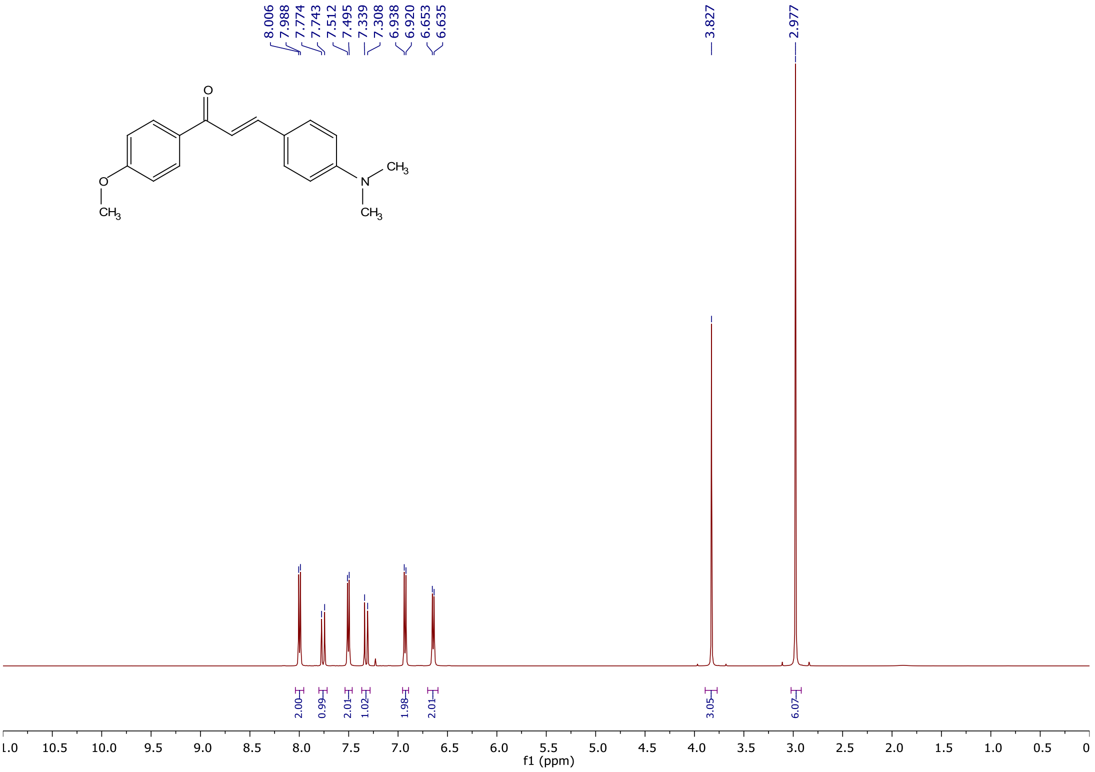

^{13}C NMR of compound **3a**

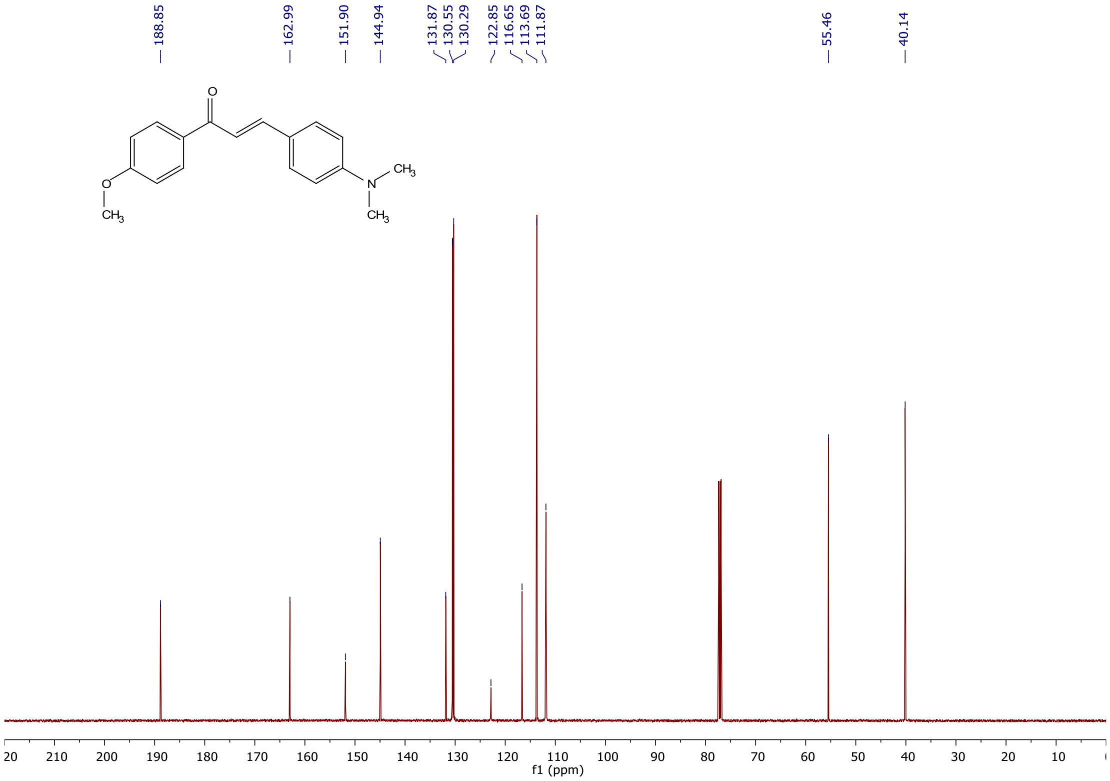

^{1}H NMR of compound **3b**

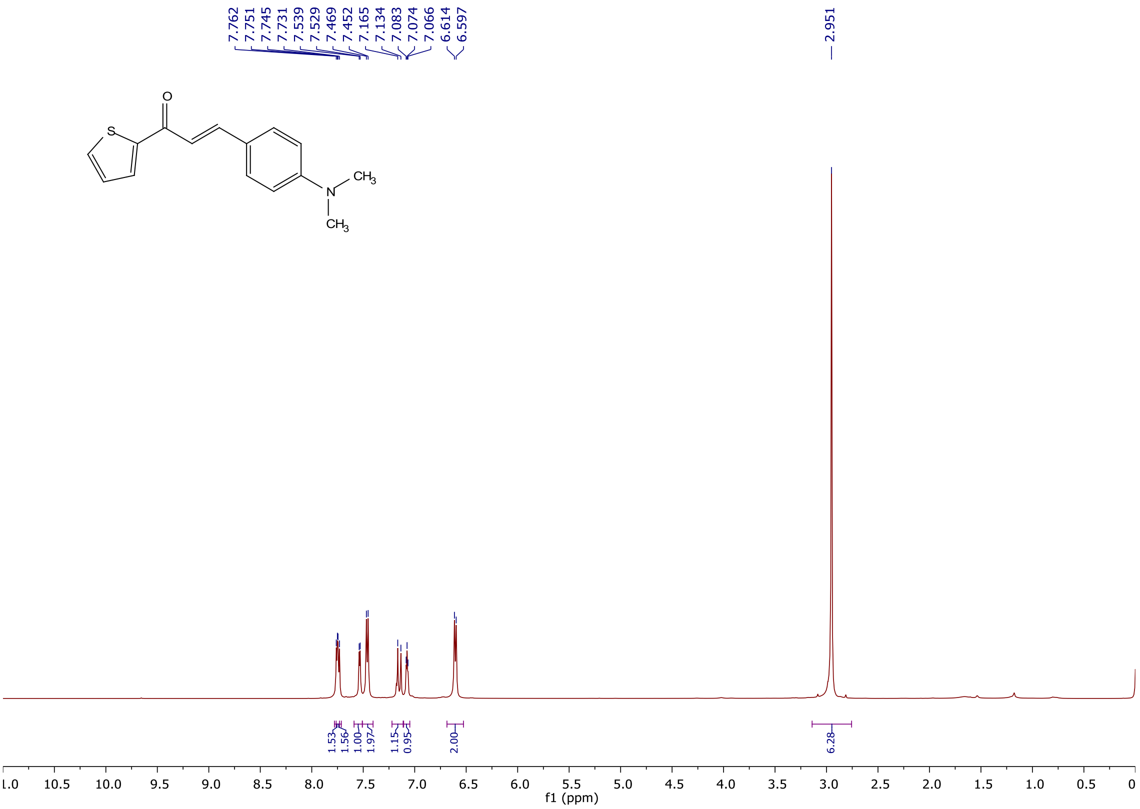

^{13}C NMR of compound **3b**

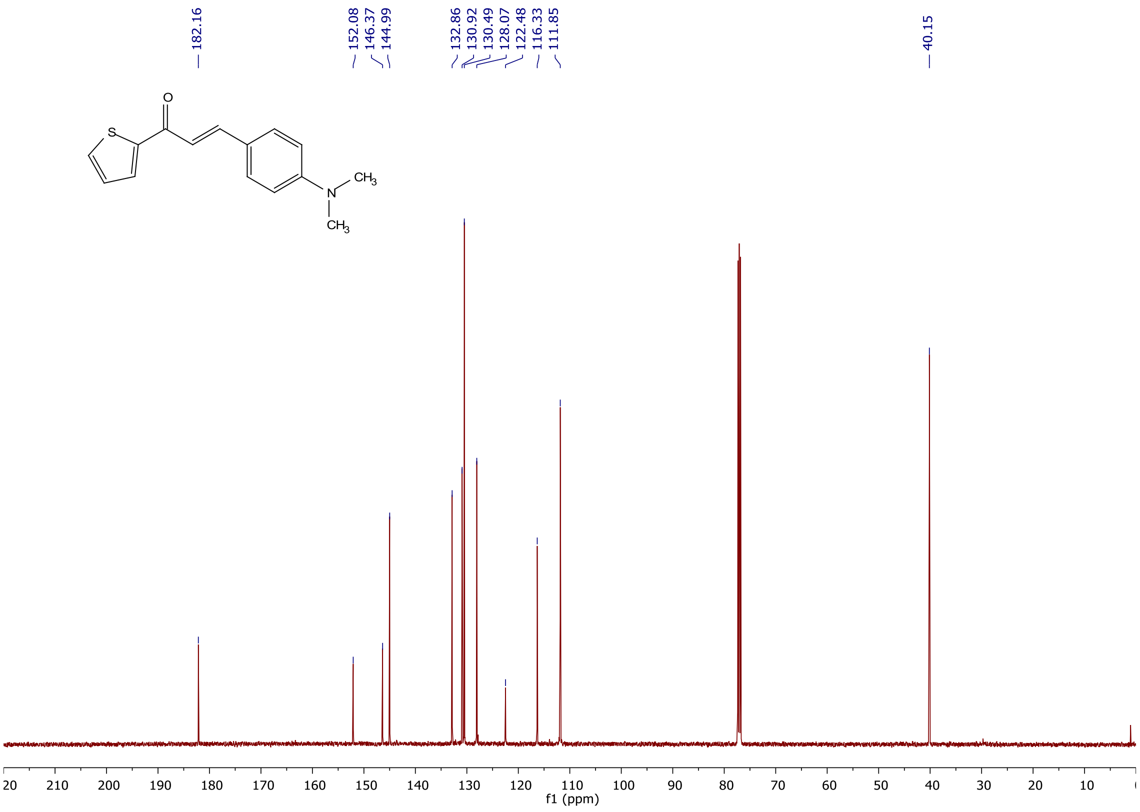

^{1}H NMR of compound **3c**

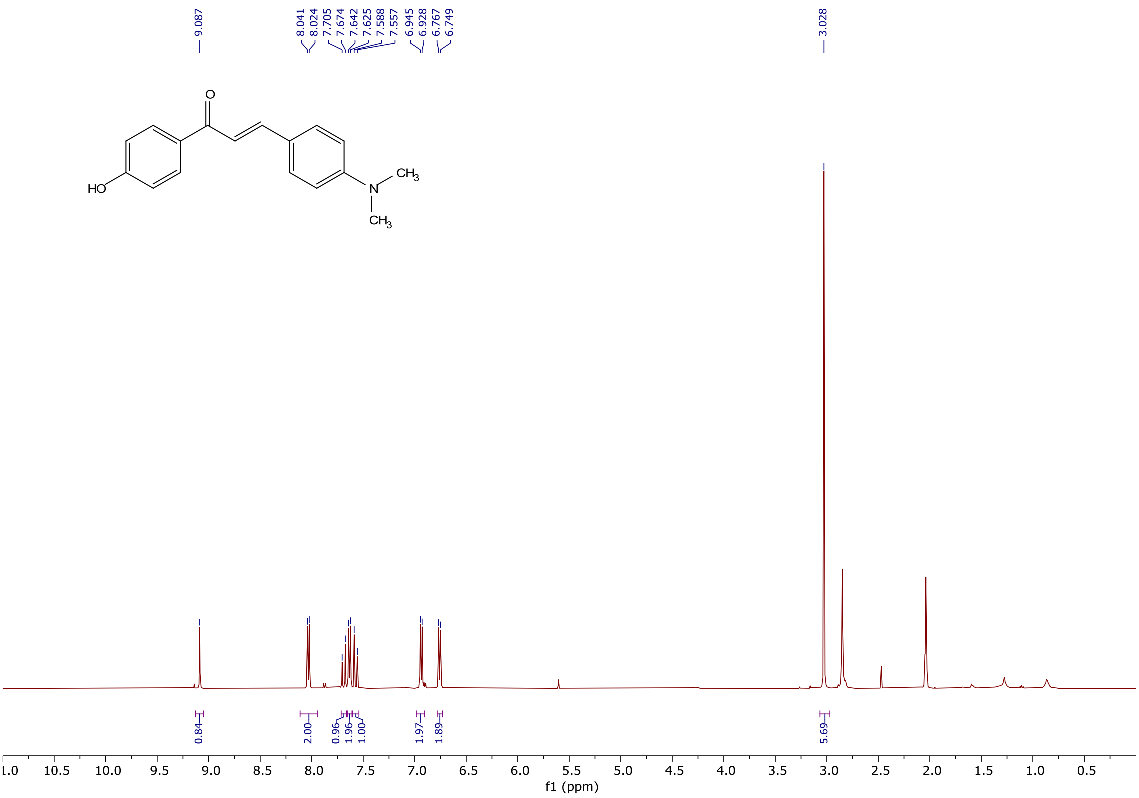

^{13}C NMR of compound **3c**

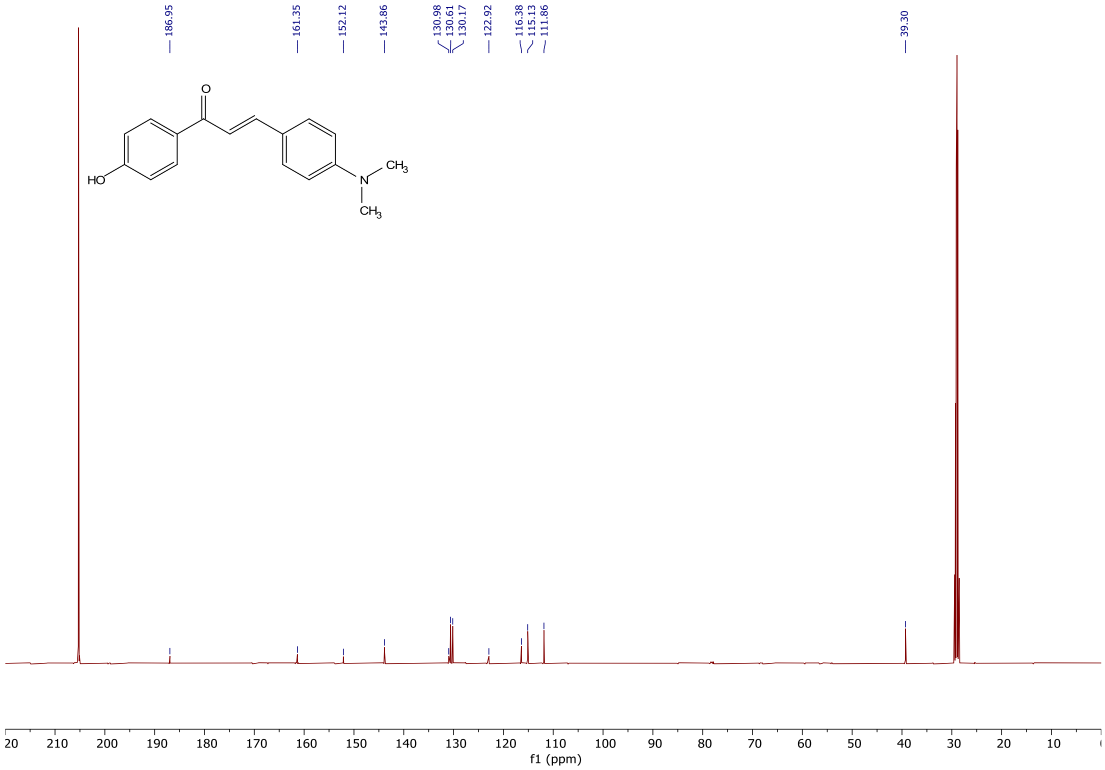

^{1}H NMR of compound **3d**

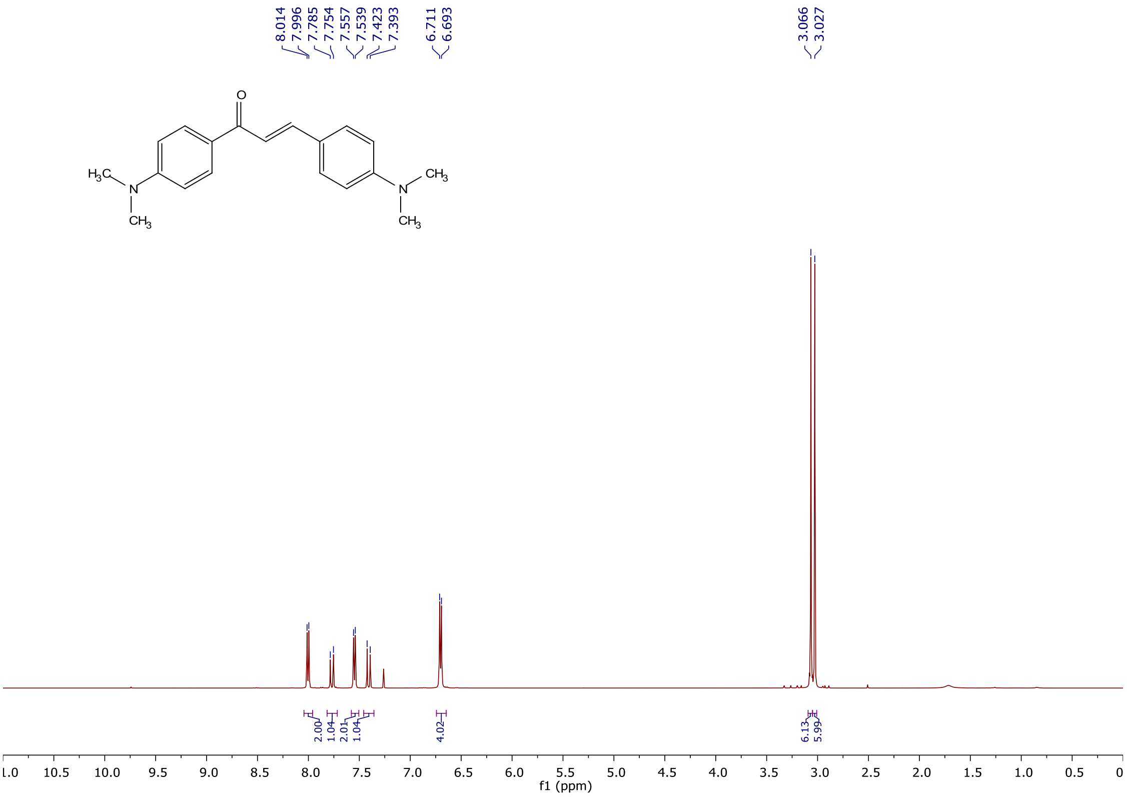

^{13}C NMR of compound **3d**

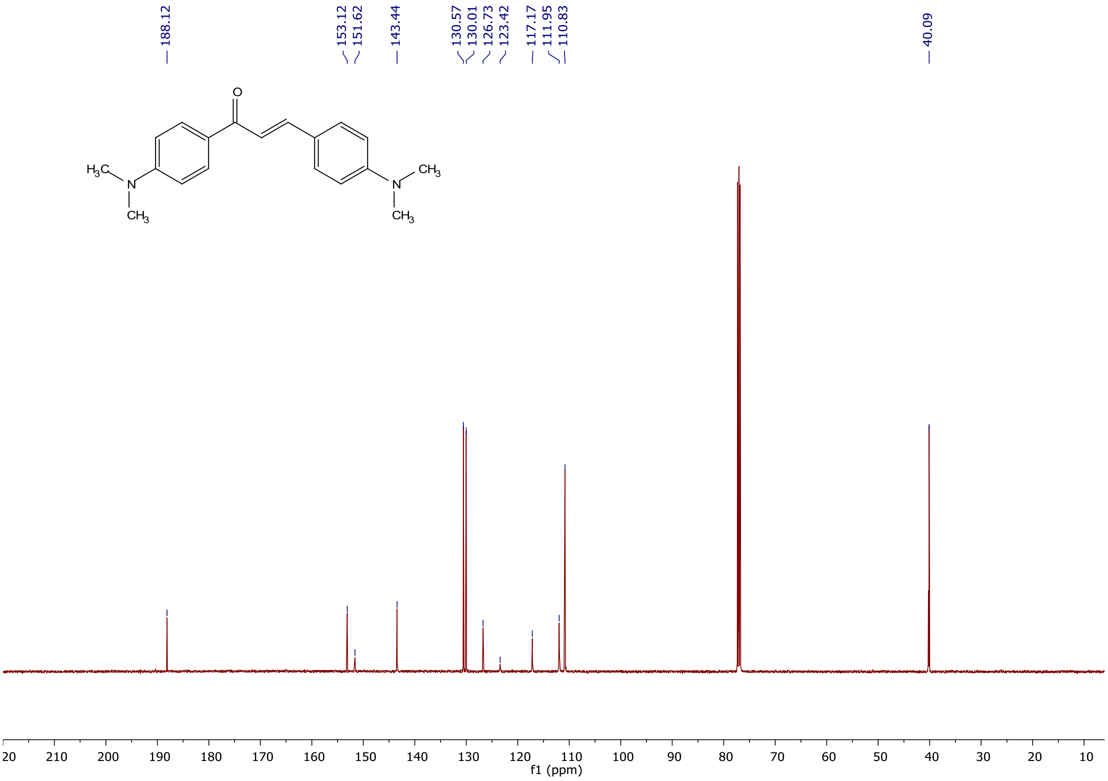

^{1}H NMR of compound **3e**

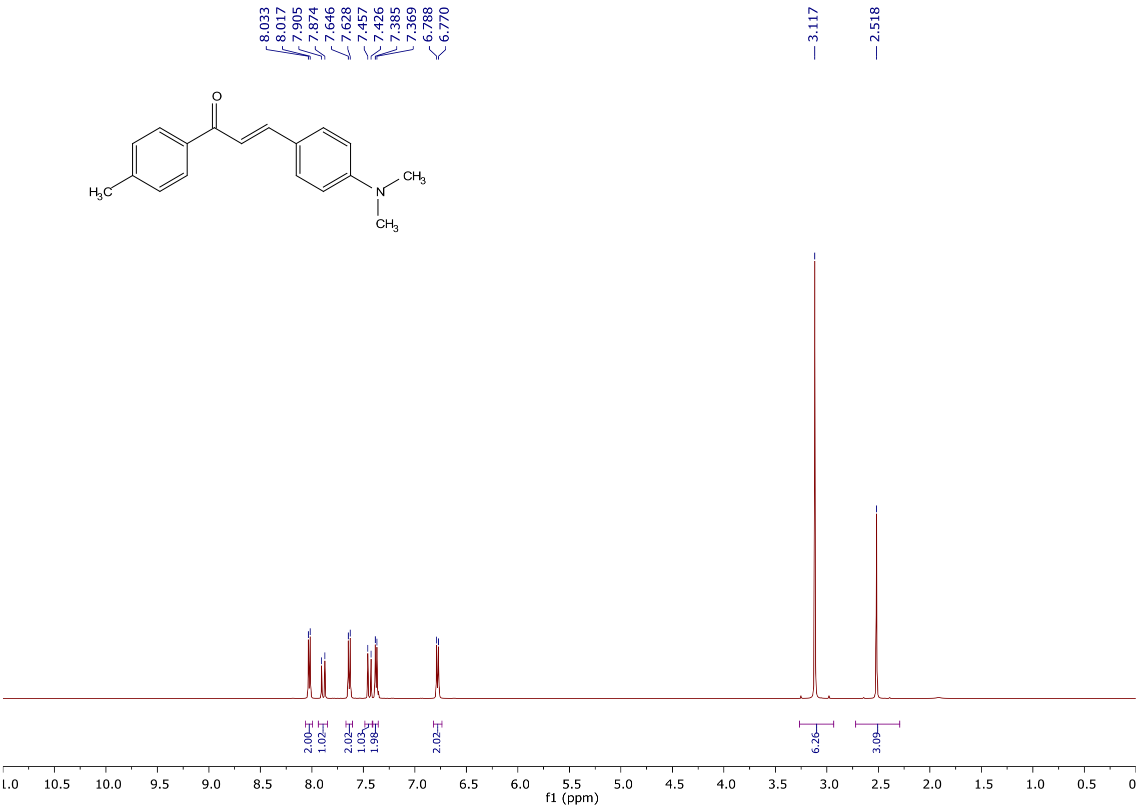

^{13} C NMR of compound **3e**

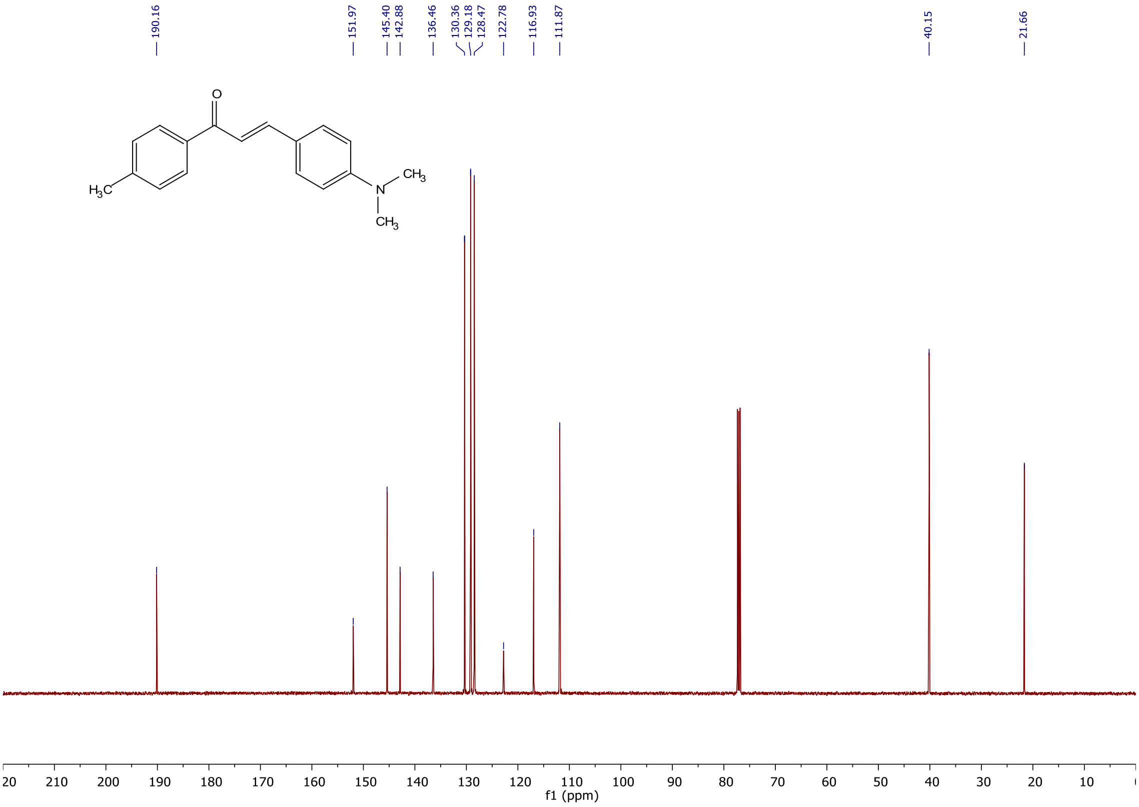

^{1}H NMR of compound **3f**

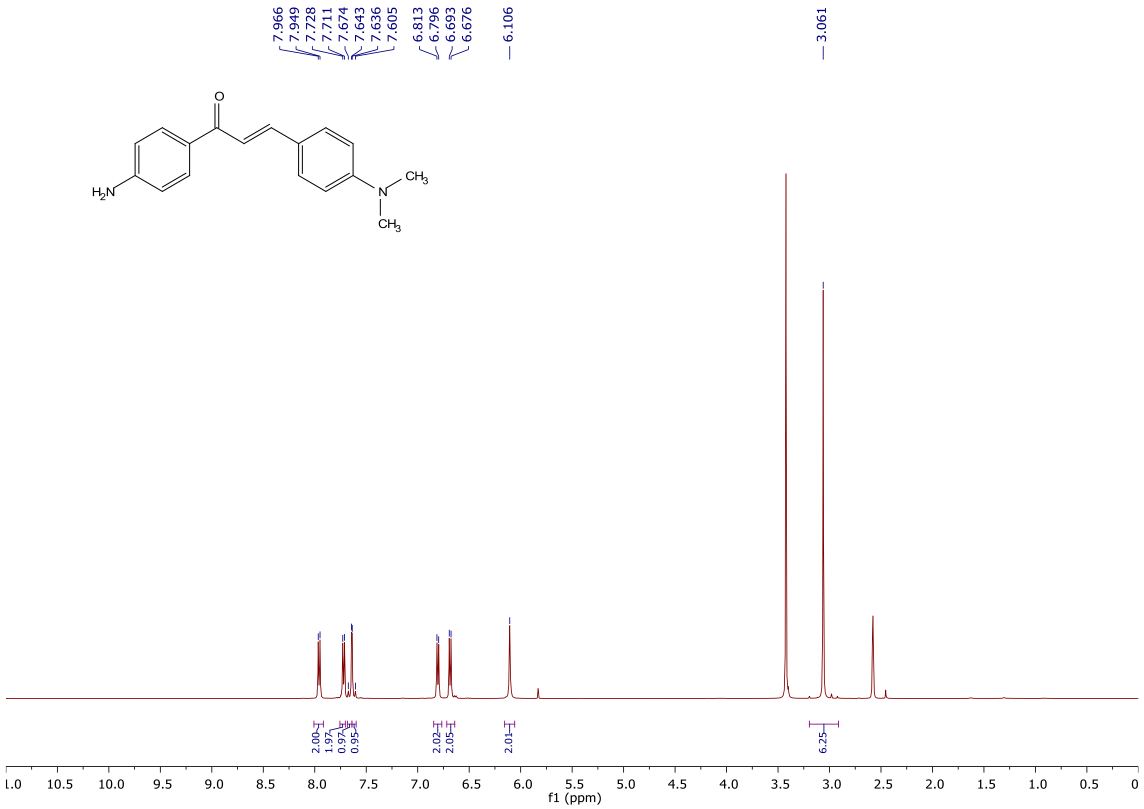

^{13}C NMR of compound **3f**

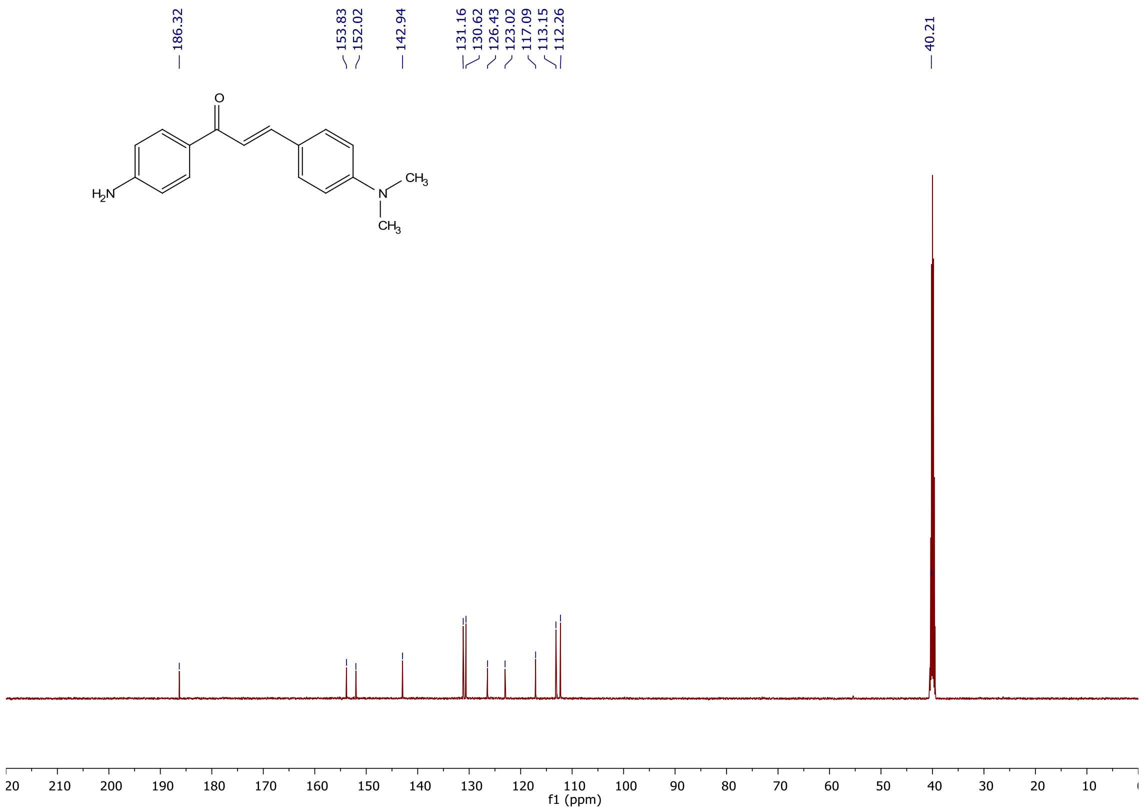

3. Absorption and fluorescence spectroscopic analyses

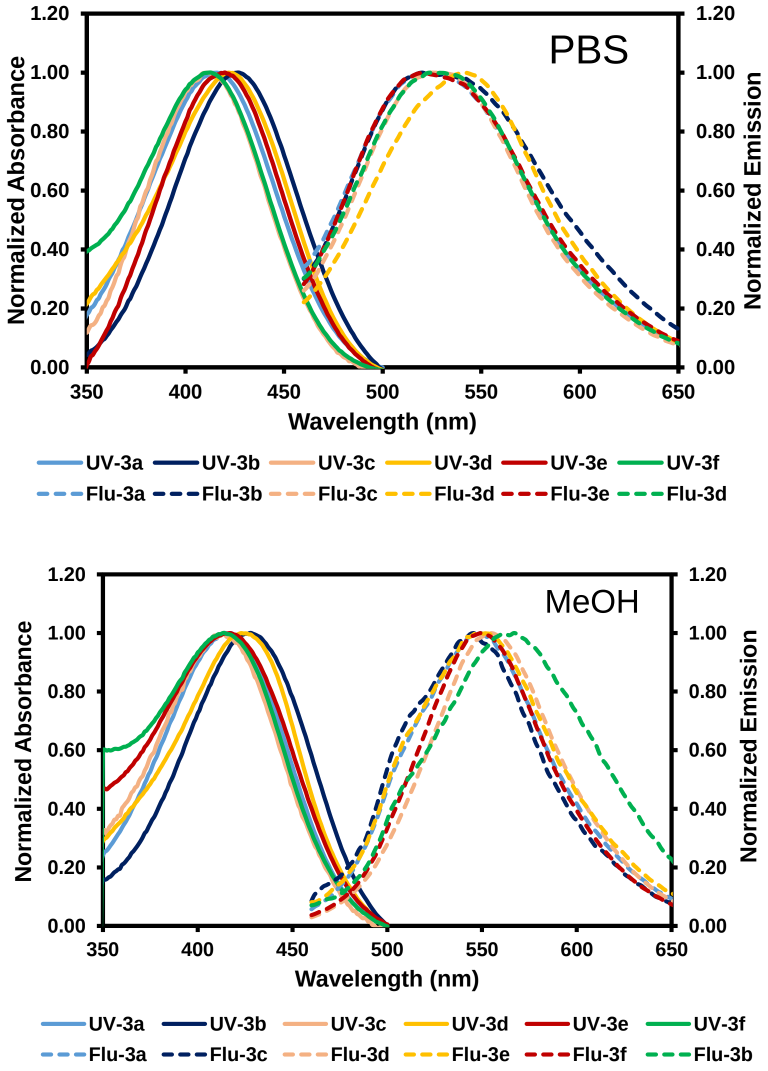

Figure S1. Normalized absorption and fluorescence spectra of chalcones in PBS and MeOH. The fluorescence spectra excited at the absorption maximum wavelength of each compound.

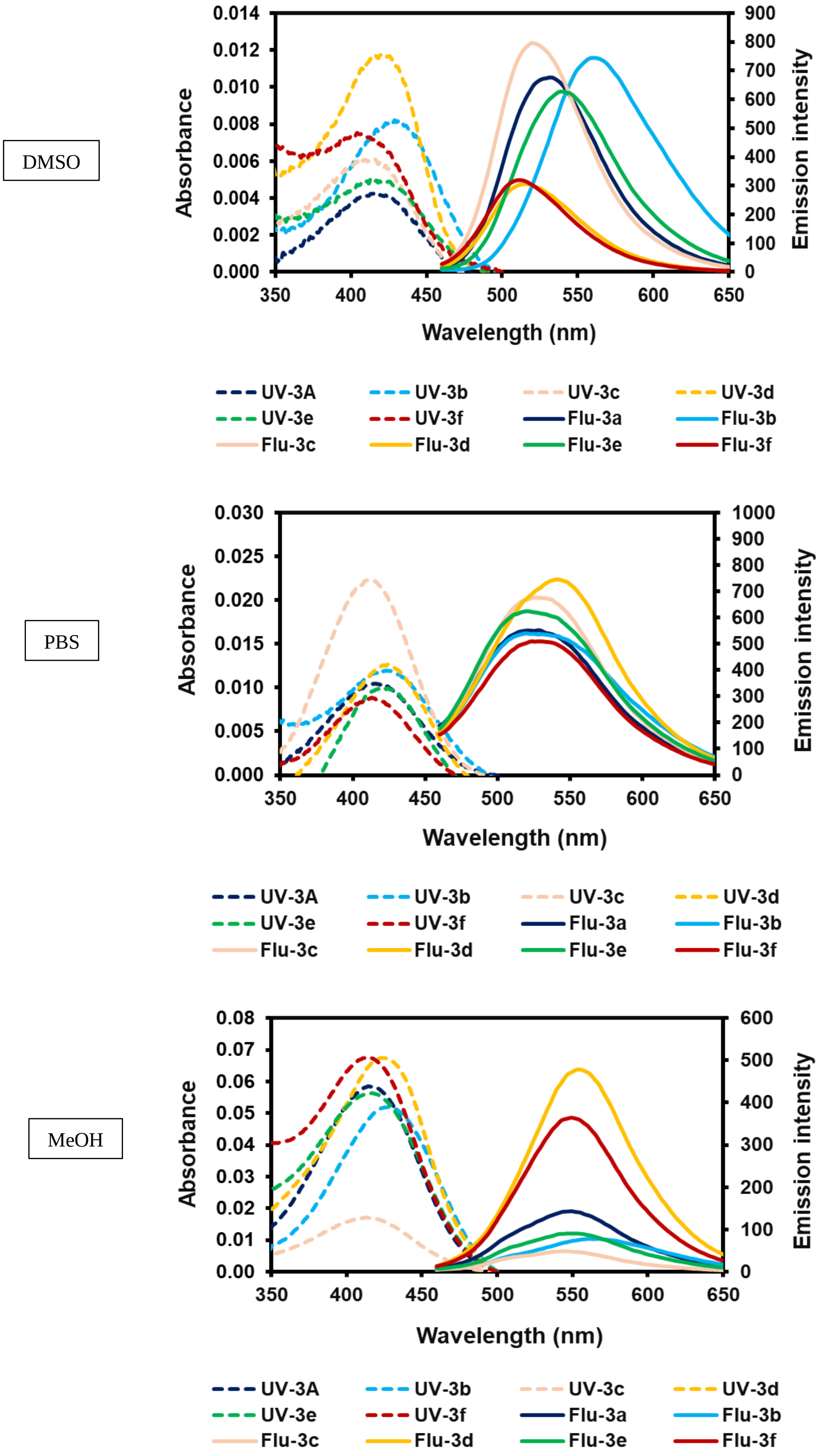

Figure S2. Absorption and fluorescence spectra (raw data) of chalcones in DMSO, PBS and MeOH. The fluorescence spectra excited at the absorption maximum wavelength of each compound.

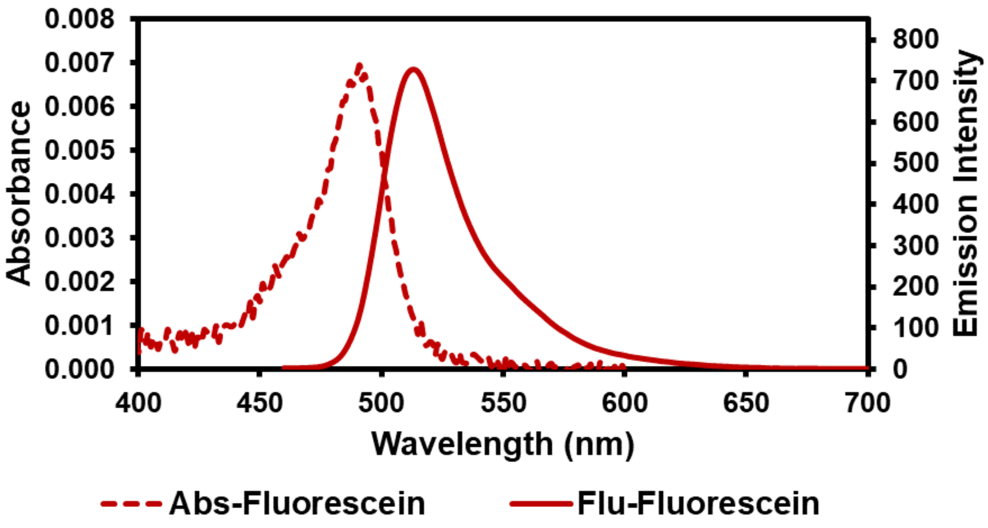

Figure S3. Absorption and fluorescence spectra of fluorescein in 0.1 M NaOH.

4. Photostability of chalcones 3a-f

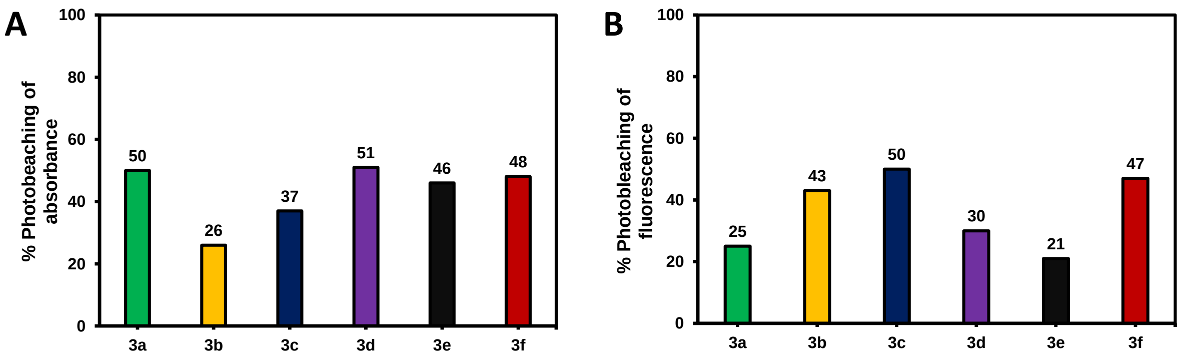

Figure S4. Calculated percentage of photobleaching of chalcones **3a-f** in DMSO based on (A) absorbance and (B) fluorescence intensity.

5. Confocal imaging of chalcones (3a-f) in HEK-293

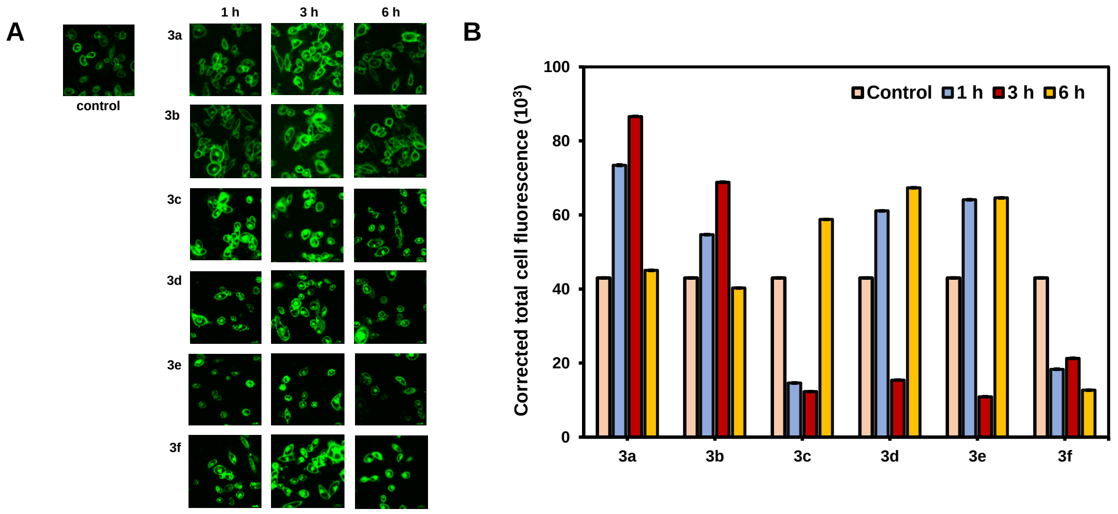

Figure S5. (A) Confocal images of HEK-293 cells incubated with chalcones (5 μM) for 1-6 h. Scale bars: 20 μm. And (B) quantitative corrected total cell fluorescence data which quantified using ImageJ and represent the mean ± SD (n = 30)

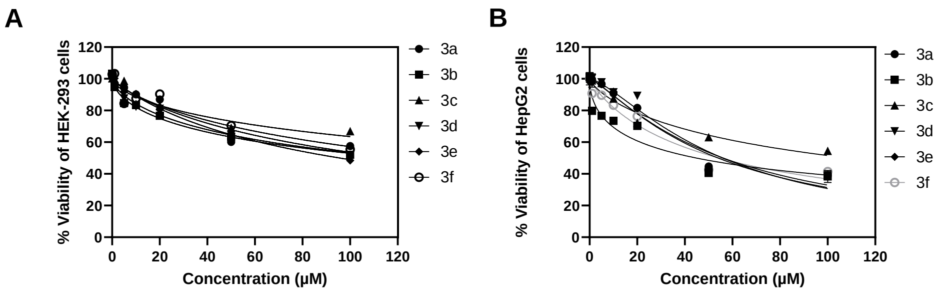

Figure S6. Plots of concentrations of **3a-f** *vs* percent viability of HEK-293 (A) and HepG2 (B) cells. Presented IC_{50} values were derived from MTT assays (n = 3).

6. Antibacterial activity of the synthesized chalcones (3a-f)

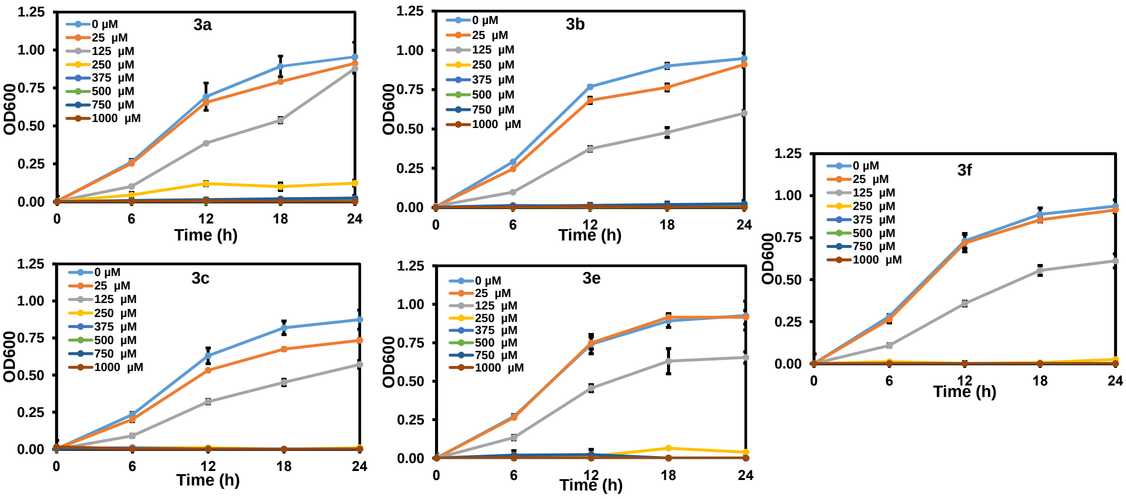

Figure S7. Growth curves of *E. coli* 780 in response to **3a-f** in a time course of 24 h.

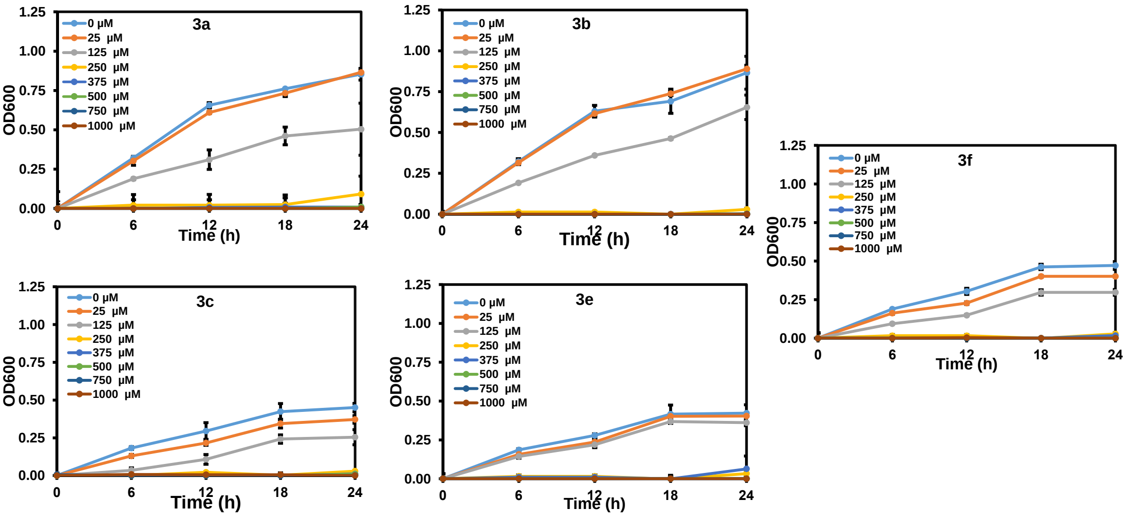

Figure S8. Growth curves of *S. aureus* 1466 in response to **3a-f** in a time course of 24 h.

Reference

(1) Syam, S.; Abdelwahab, S. I.; Al-Mamary, M. A.; Mohan, S. *Molecules* **2012**, *17*, 6179.

(2) Kar, S.; Mishra, R. K.; Pathak, A.; Dikshit, A.; Golakoti, N. R. *J. Mol. Struct.* **2018**, *1156*, 433.

(3) Kar, S.; Adithya, K. S.; Shankar, P.; Jagadeesh Babu, N.; Srivastava, S.; Nageswara Rao, G. *J. Mol. Struct.* **2017**, *1139*, 294.

(4) Bhatia, N. M.; Mahadik, K. *Sci. Pharm.* **2008**, *76*, 259.

(5) Prasad, Y. R.; Rani, V. J.; Rao, A. S. *Asian J. Chem.* **2013**, *25*, 52.
